# Backache, Sciatica and Ruptured Discs
*An address to the Torquay and District Medical Society at the Torbay Hospital, on Thursday, 25th January, 1951.


**Published:** 1951-07

**Authors:** Kenneth Pridie

**Affiliations:** Orthopaedic Surgeon, United Bristol Hosptals


### BACKACHE, SCIATICA AND RUPTURED DISCS*

BY

KENNETH PRIDIE, F.R.C.S.

*Orthopaedic Surgeon, United Bristol Hosptals*

---

Mr. Pridie prefaced his address by saying that his title would appear to cover a comparatively wide field. Yet what he really proposed was to discuss the relationship of disc disturbances to backache and sciatica, and to demonstrate that certain disc disturbances give rise to a definite clinical picture. Showing a number of slides and drawings explaining the development and anatomical position of the nucleus pulposus and its surrounding structures, he demonstrated that sudden and unexpected or unusual forces will cause weakness or rupture of the annulus fibrosus, and the consequent protusion of the nucleus through the affected part of the annulus. This occurs mainly posteriorly and just to the right or left of the midline. Any degree of protusion may occur, from a mild bulge to almost complete extrusion of the nucleus. This leads to pressure on the dura itself or on the nerve roots as they leave the dura. The signs and symptoms of this condition are reasonably clear-cut. The patient has the back locked in a position of flexion or extension: and when examined in an upright posture and asked to bend forward, when the back is fixed in extension, no flexion takes place in the lumbar region. The lumbar region is maintained stiff and he bends to the right or left, causing a scoliosis away from the side of the affection (this point was illustrated by lantern slides and a most instructive film). The condition may

* An address to the Torquay and District Medical Society at the Torbay Hospital, on Thursday, 25th January, 1951.

be confirmed by mapping out in the affected leg definite areas of altered sensation (paraesthesia), depending on the segmental nerves involved. There is nearly always a history of unwonted or unusual exercise or a twist or strain, which was followed by acute pain in the back, and is associated with sciatica which may come on immediately or at a later date.

For the acute cases treatment consists first of rest in bed for a month followed by the prohibition of heavy lifting or hard work for a further month. If this fails, then more rigid immobilization becomes essential and the patient should be immobilized in a plaster jacket for at least a month or six weeks. If then the patient is no better operation should be considered. Chronic cases or cases seen late are best treated by manipulation. It is preferable to do this without an anaesthetic and the manipulation should always be in a position of flexion. In some cases this will give immediate relief as it relieves the pressure on the nerve roots.

Skiagrams should always be taken when the condition is one of slowly progressive pain, rather than an acute sudden attack coming on after an accident, in order to eliminate any other pathological changes in the bone. It was emphasized that the osteophytic spurs seen around the edge of the vertebrae were indications of old lesions and often had no connection with the condition of which the patient is complaining. Some indirect evidence, however, may be obtained by noticing the narrowing of the intervertebral joint spaces posteriorly, particularly between Lumbar 5 and Sacral 1. A dead straight back in an X-ray, that is, complete loss of the lumbar curve, is an indication of a disc lesion.

The contra-indications to manipulation are pathological changes showing in an X-ray film, slow development of the pain and deformity or if straight-leg-raising is markedly diminished. If manipulation is undertaken in these cases further disc protrusion may be caused. Manipulation should also not be undertaken where there are marked neurological signs, such as loss of ankle or knee reflexes, weakness of the extensors, peronei muscles or quadriceps. The indications for manipulation would be that the patient has had relief from this method of treatment before, or a chronic case where the clinical signs are not very marked and where there is no sign of a tension lesion.

If all the above methods of treatment fail then operation should be considered and the indications for this are:

(*a*) Failure of conservative treatment to relieve pain.

(*b*) Severe and persistent pain with definite physical signs.

(*c*) Well-marked neurological signs; particularly diminished straight-leg-raising, diminished or lost ankle jerks, and weakness of muscles. It has been observed that a completely lost ankle jerk is seldom recovered whereas a diminished ankle jerk will return to normal in about a year.

